# Self-Excited Microcantilever with Higher Mode Using Band-Pass Filter

**DOI:** 10.3390/s23052849

**Published:** 2023-03-06

**Authors:** Yuji Hyodo, Hiroshi Yabuno

**Affiliations:** Degrees Programs in Systems and Information Engineering, Graduate School of Science and Technology, University of Tsukuba, Tsukuba 305-8573, Ibaraki, Japan

**Keywords:** MEMS, microcantilever, self-excited oscillation, higher mode

## Abstract

Microresonators have a variety of scientific and industrial applications. The measurement methods based on the natural frequency shift of a resonator have been studied for a wide range of applications, including the detection of the microscopic mass and measurements of viscosity and stiffness. A higher natural frequency of the resonator realizes an increase in the sensitivity and a higher-frequency response of the sensors. In the present study, by utilizing the resonance of a higher mode, we propose a method to produce the self-excited oscillation with a higher natural frequency without downsizing the resonator. We establish the feedback control signal for the self-excited oscillation using the band-pass filter so that the signal consists of only the frequency corresponding to the desired excitation mode. It results that careful position setting of the sensor for constructing a feedback signal, which is needed in the method based on the mode shape, is not necessary. By the theoretical analysis of the equations governing the dynamics of the resonator coupled with the band-pass filter, it is clarified that the self-excited oscillation is produced with the second mode. Furthermore, the validity of the proposed method is experimentally confirmed by an apparatus using a microcantilever.

## 1. Introduction

Microresonators have a variety of scientific and industrial applications [1]. The measurement methods based on the natural frequency shift of a resonator or an actuator have been studied for a wide range of applications, including the detection of the microscopic mass and measurements of viscosity [2,3,4,5] and stiffness [6] and many others physical quantities [7]. Reducing the dimensions of the resonator increases the natural frequency of a resonator and achieves a high sensitivity and a high-frequency response in measurements. However, there is a limitation in manufacturing to construct an extremely downsized resonator. Moreover, in the downsized resonator, it is very difficult to coat the chemical receptor because of the small surface. Thus, methods to increase the natural frequency are expected without downsizing the resonator. An effective method is to utilize the natural frequency of the higher mode in beam-type resonators, which is larger than the fundamental natural frequency [8,9]. Ghatkesar et al. showed that the sensitivity of a mass measurement using the natural frequency shift of a cantilever-type resonator is proportional to the square of the order number of the modes [10]. Dohn et al. showed that the natural frequency shift of a cantilever vibrating in the fourth-order mode provides a high resolution in the measurement not only for the mass of the attached particle but also for its position [11]. By the way, there are mainly two methods to detect the natural frequency of a resonator, which are by the application of external or forced excitation and by using self-excitation based on positive velocity feedback control. There is also a third method where an actuator of piezoelectrical crystal is utilized as a resonator. When detecting physical quantities, the dynamics of the actuator are modified as shown by the equivalent circuit [7]. In the first method, the natural frequency is detected from the excitation frequency at which the frequency response curve has a peak. This method makes it easy to detect the natural frequency of a higher mode by applying the external excitation frequency in the neighborhood of the natural frequency of the higher mode. However, in viscous environments, the excitation frequency at the peak of the frequency response curve deviates from the desired natural frequency. Moreover, in much higher viscous damping, the resonance peak itself does not exist; the external excitation method is not applicable to the detection of a natural frequency. To overcome this difficulty, the second method using self-excitation is applicable, where the viscous damping effect is compensated for by positive velocity feedback [12,13,14,15,16]. The velocity or displacement to establish the feedback is measured at a point of the beam-type resonator and the measurement signal may generally include the natural frequencies for many modes. In order to achieve the self-excited oscillation with a higher mode, Zhou et al. set the sensor position so that the measurement signal included a smaller frequency component of lower modes than the targeted one and experimentally realized the self-excited oscillation with the second natural frequency [17].

In this study, we establish the feedback control signal for the self-excited oscillation using the band-pass filter so that the signal consists of only the frequency corresponding to the desired excitation mode. We analyze the discretized equations of motion of a cantilever, which consider the first two modes, which are coupled with the circuit equation of a band-pass filter. Then, we reveal using root loci that the self-excited oscillation of the second mode can be selectively generated by matching the center frequency of the band-pass filter with that of the second mode. We implement the proposed method with a microcantilever and experimentally confirm the validity of the proposed method by carrying out the self-excited oscillation with the second mode.

## 2. Method to Produce Self-Excited Oscillation with Higher Mode in Cantilever Beam

### 2.1. Analytical Model of the Cantilever Subject to a Base Excitation

**Figure 1:**
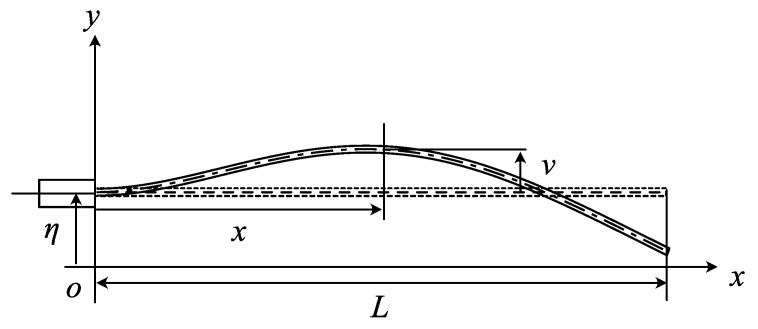
Analytical model of cantilever subject to the displacement excitation; η is the displacement excitation by actuator to produce the self-excited oscillation.

The analytical model of the cantilever beam is shown in Figure 1, where *L* and *v* are the cantilever length and the displacement in the lateral direction, respectively.

We assume that the cantilever behaves as a uniform linear Euler–Bernoulli beam. The equation of motion and the associated boundary conditions are expressed as

(1) $$\rho A\frac{\partial^{2}}{\partial t^{2}}\left(v+\eta \right)+c\frac{\partial }{\partial t}v+EI\frac{\partial^{4}}{\partial x^{4}}v=0,$$

(2) $${v|}_{x=0}=\frac{\partial }{\partial x}{v|}_{x=0}=\frac{\partial^{2}}{\partial x^{2}}v{{|}_{x=L}=\frac{\partial^{3}}{\partial x^{3}}v|}_{x=L}=0,$$

where ρA is the mass per unit length, *c* is the coefficient of viscous damping proportional to the velocity, and EI is the bending stiffness of the cantilever.

We introduce the nondimensionalized coordinate $x^{*}=\frac{1}{L}x$, the nondimensionalized displacement $v^{*}=\frac{1}{L}v$, the nondimensionalized displacement excitation $\eta^{*}=\frac{1}{L}\eta$, and the nondimensionalized time $t^{*}=\frac{1}{T}t$, where $T=L^{2}\sqrt{\frac{\rho A}{EI}}$ is the representative time. The nondimensionalized equation of motion of the cantilever beam and the nondimensionalized boundary conditions are derived as follows:

(3) $${\ddot{v}}^{*}+2\mu^{*}{\dot{v}}^{*}+v^{*\prime \prime \prime \prime }=-{\ddot{\eta }}^{*},$$

(4) $$v^{*}{|}_{x^{*}=0}=v^{*\prime }{|}_{x^{*}=0}=v^{*\prime \prime }{{|}_{x^{*}=1}=v^{*\prime \prime \prime }|}_{x^{*}=1}=0,$$

where $2\mu^{*}=\frac{cL^{2}}{\sqrt{\rho AEI}}$ is the nondimensionalized damping coefficient, and [˙] and [${\;}^{\prime }$] denote $\frac{\partial }{\partial t^{*}}$ and $\frac{\partial }{\partial x^{*}}$, respectively.

We express $v^{*}$ considering the first and second modes as [18,19]

(5) $$v^{*}\left(t^{*},x^{*}\right)=v_{1}^{*}\left(t^{*}\right)\Phi_{1}^{*}\left(x^{*}\right)+v_{2}^{*}\left(t^{*}\right)\Phi_{2}^{*}\left(x^{*}\right).$$

where $v_{i}^{*}\left(t^{*}\right)$ is the *i*th modal coordinate. $\Phi_{i}^{*}\left(x^{*}\right)$ is the *i*th modal function, which satisfies

(6) $$\Phi_{i}^{*\prime \prime \prime \prime }-\omega_{i}^{*2}\Phi_{i}^{*}=0,$$

and it is normalized to satisfy

(7) $$\frac{\int_{0}^{1}\Phi_{i}^{*2}dx^{*}}{\int_{0}^{1}\Phi_{i}^{*}dx^{*}}=1,$$

where $\omega_{i}^{*}$ is the nondimensionalized *i*th natural frequency. The boundary conditions of the *i*th modal function are

(8) $$\Phi_{i}^{*}{|}_{x^{*}=0}=\Phi_{i}^{*\prime }{|}_{x^{*}=0}=\Phi_{i}^{*\prime \prime }{{|}_{x^{*}=1}=\Phi_{i}^{*\prime \prime \prime }|}_{x^{*}=1}=0.$$

From Equations (6) and (8), the natural frequency $\omega_{i}^{*}$ satisfies

(9) $$\cos\sqrt{\omega_{i}^{*}}\cosh\sqrt{\omega_{i}^{*}}=-1.$$

Substituting Equation (5) into Equation (3) and applying the orthogonality of the mode functions, we obtain the discretized equations of motion for the first and second modes as follows:

(10) $$\begin{array}{c} \left\{\begin{array}{c} \ddot{v_{1}^{*}}+2\mu^{*}\dot{v_{1}^{*}}+\omega_{1}^{*}v_{1}^{*}=-\ddot{\eta^{*}}\\ \ddot{v_{2}^{*}}+2\mu^{*}\dot{v_{2}^{*}}+\omega_{2}^{*}v_{2}^{*}=-\ddot{\eta^{*}}. \end{array}\right. \end{array}$$

To generate self-excited oscillation by compensating for the effect of viscous damping, we apply the base excitation $\eta^{*}$ based on the integral feedback control with respect to the displacement of the cantilever in the nondimensionalized form as

(11) $$\eta^{*}=\alpha^{*}\int v^{*}{|}_{x^{*}=x_{m}^{*}}dt,$$

where $\alpha^{*}$ is the feedback gain and $x_{m}^{*}$ is the nondimensionalized horizontal position of the measurement point. Substituting Equation (11) into Equation (10) yields

(12) $$\begin{array}{c} \left\{\begin{array}{c} \ddot{v_{1}^{*}}+\left(2\mu^{*}+\alpha^{*}a_{1}^{*}\right)\dot{v_{1}^{*}}+\omega_{1}^{*}v_{1}^{*}=-\alpha^{*}a_{2}^{*}\dot{v_{2}^{*}}\\ \ddot{v_{2}^{*}}+\left(2\mu^{*}+\alpha^{*}a_{2}^{*}\right)\dot{v_{2}^{*}}+\omega_{2}^{*}v_{2}^{*}=-\alpha^{*}a_{1}^{*}\dot{v_{1}^{*}}, \end{array}\right. \end{array}$$

where $a_{i}^{*}=\Phi_{i}^{*}\left(x_{m}^{*}\right)$. As seen from Equation (12), the integral feedback acts on the systems as velocity feedback. When the feedback gain $\alpha^{*}$ satisfies

(13) $$\alpha^{*}<-\frac{2\mu^{*}}{a_{i}^{*}},$$

the *i*th mode is self-excited. The critical feedback gains for the self-excited oscillations are different depending on the modes because $a_{i}^{*}$ is determined by the mode shape $\Phi_{i}^{*}$. To selectively produce the self-excited oscillation with only a specified mode, suitably setting the relation between $a_{i}^{*}$ is necessary. For example, in order to produce the self-excited oscillation with the second mode, the critical feedback gains for the first and second modes, $\alpha_{1cr}^{*}=-\frac{2\mu^{*}}{a_{1}^{*}}$ and $\alpha_{2cr}^{*}=-\frac{2\mu^{*}}{a_{2}^{*}}$, have to satisfy $\alpha_{2cr}^{*}<\alpha_{1cr}^{*}$. To this end, Zhou et al. set the sensor position so that the measurement signal included a smaller frequency component of lower modes than the targeted one [17]. This method is necessary to accurately adjust the measurement point in practice. In the present study, in order to avoid such adjustment, by using a band-pass filter, we propose a method to produce the self-excited oscillation with a desired mode.

### 2.2. Genaration of Self-Excited Oscillation in a Higher Mode Using Band-Pass Filter

We apply a filter to the feedback signal to attenuate the frequency components of modes other than the target one. To eliminate undesired frequency components in the signal, high-pass or low-pass filters are generally used, but a signal passed through such a filter has a phase difference depending on the frequency components from the original one. Many sensors using the natural frequency shift of a resonator have been proposed as mass sensors, stiffness sensors, atomic force microscopes, and so on. The measurement accuracy of the natural frequency is directly related to that of the sensors. By the feedback of Equation (11), the proposed method compensates for the viscous damping and produces the self-excited oscillation. Therefore, the phase of the actuation η has to be equal to that of the velocity of the cantilever. If a phase difference exists, the response frequency in the self-excited cantilever deviates from the original natural frequency of the cantilever due to the effect of feedback and the accuracy of the natural frequency detection is degraded. In this study, we utilize a band-pass filter so as not to generate such a phase difference and also to attenuate the undesired frequency component in the actuation signal $\eta^{*}$. A second-order band-pass filter can be constructed by combining first-order low-pass and high-pass filters in series. The input voltage $V_{in}$ to the band-pass filter corresponds to the left-hand side of Equation (11) and is calculated in real-time using the measured displacement $v(t,x_{m})$ in the practical system according to the following:

(14) $$V_{in}=g_{in}\int {v|}_{x=x_{m}}dt,$$

where $g_{in}$ is the gain of the input voltage, and $x_{m}$ is the measurement point for the displacement of the cantilever beam. The output voltage $V_{out}$ is used to the excitation η as

(15) $$\eta =g_{actuator}V_{out},$$

where $g_{actuator}$ is the piezoelectric constant of the piezo actuator, which denotes the strain of the piezo actuator per electric field. The relationship between the input and output voltages, $V_{in}$ and $V_{out}$, is expressed as

(16) $$\frac{d^{2}}{dt^{2}}V_{out}+\left(\omega_{L}+\omega_{H}\right)\frac{d}{dt}V_{out}+\omega_{L}\omega_{H}V_{out}=\left(\omega_{L}+\omega_{H}\right)\frac{d}{dt}V_{in},$$

where $\omega_{L}$ and $\omega_{H}$ are the cutoff frequencies of the low-pass and high-pass characteristics, respectively; $BW=\omega_{L}+\omega_{H}$ is the bandwidth. The derivation of Equation (16) is shown in Appendix A. When the input frequency equals the center frequency $\omega_{c}=\sqrt{\omega_{L}\omega_{H}}$, the amplitude gain and the phase difference between the input and output voltages are 1 and 0, respectively.

Substituting Equation (14) into Equations (10) and (16) and introducing nondimensionalized time $t^{*}$ and nondimensionalized voltage $V_{out}^{*}=\frac{1}{V}V_{out}$, where the representative voltage $V=\frac{L}{g_{actuator}}$, we obtain the nondimensionalized equations governing the dynamics of the first and second modes of the cantilever coupled with the band-pass filter under feedback control as

(17) $$\begin{array}{c} \left\{\begin{array}{c} {\ddot{v_{1}}}^{*}+2\mu^{*}{\dot{v_{1}}}^{*}+\omega_{1}^{*}{v_{1}}^{*}=-{\ddot{V}}_{out}^{*}\\ {\ddot{v_{2}}}^{*}+2\mu^{*}{\dot{v_{2}}}^{*}+\omega_{2}^{*}{v_{2}}^{*}=-{\ddot{V}}_{out}^{*}\\ {\ddot{V}}_{out}^{*}+2\beta^{*}\omega_{c}^{*}{\dot{V}}_{out}^{*}+\omega_{c}^{*2}V_{out}^{*}=2\beta^{*}\omega_{c}^{*}\alpha^{*}\left(a_{1}^{*}v_{1}^{*}+a_{2}^{*}v_{2}^{*}\right), \end{array}\right. \end{array}$$

where $\beta^{*}=\frac{\omega_{L}+\omega_{H}}{2\omega_{c}}$ is the nondimensionalized bandwidth, $\omega_{c}^{*}=T\omega_{c}$ is the nondimensionalized center frequency, $\alpha^{*}=g_{actuator}g_{in}T$ is the feedback gain, and $a_{i}^{*}=\Phi_{i}^{*}\left(x_{m}^{*}\right)$.

From the Laplace transformation of Equation (17), we obtain the characteristic equation as

(18) $$\begin{array}{c} \left(s^{2}+2\mu^{*}s+\omega_{1}^{*2}\right)\left(s^{2}+2\mu^{*}s+\omega_{2}^{*2}\right)\left(s^{2}+2\beta^{*}\omega_{c}^{*}s+\omega_{c}^{*2}\right)\\ \;\;\;\;\;\;\;\;\;\;\;\;+2\alpha^{*}\beta^{*}\omega_{c}^{*}\left\{a_{1}^{*}s^{2}\left(s^{2}+\mu^{*}s+\omega_{1}^{*2}\right)+a_{2}^{*}s^{2}\left(s^{2}+\mu^{*}s+\omega_{2}^{*2}\right)\right\}=0. \end{array}$$

**Figure 2:**
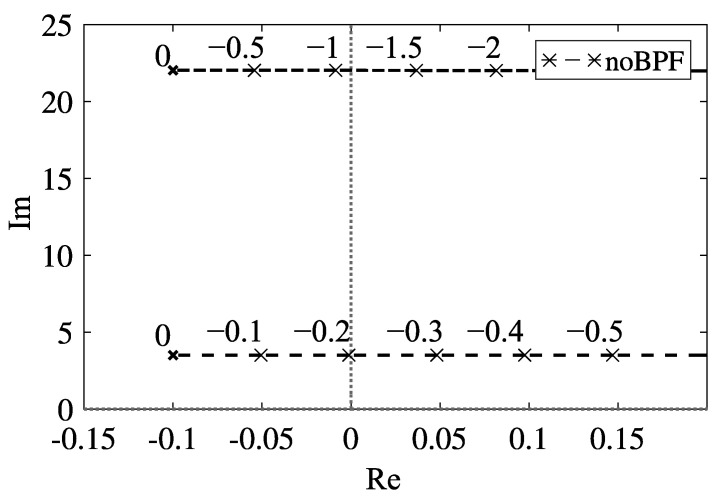
Root locus with respect to the feedback gain $\alpha^{*}$ without band-pass filter.

The root loci corresponding to the first and the second modes are shown in Figure 2; the parameters used in the numerical calculations are listed in Table 1. To examine the relationship between the center frequency and root loci, using the weight parameter *e*, we express the center frequency $\omega_{c}^{*}$ as

(19) $$\omega_{c}^{*}=\left(1-e\right)\omega_{1}^{*}+e\omega_{2}^{*}.$$

The dashed black lines with the cross marker in Figure 2 represent the root locus with respect to the variation of feedback gain $\alpha^{*}$ in Equation (11). Without the band-pass filter, the critical feedback gains of the first and second modes are $\alpha_{1cr}^{*}=-0.203$ and $\alpha_{2cr}^{*}=-1.095$, respectively. Because the absolute value of $\alpha_{1cr}^{*}$ is smaller than that of $\alpha_{2cr}^{*}$, the self-excited oscillation occurs with the first mode in the case without the band-pass filter. Therefore, to realize the self-excited oscillation with the first mode, we do not need the band-pass filter. Thus, we consider the utilization of the band-pass filter to produce the self-excited oscillation with the second mode.

**Figure 3:**
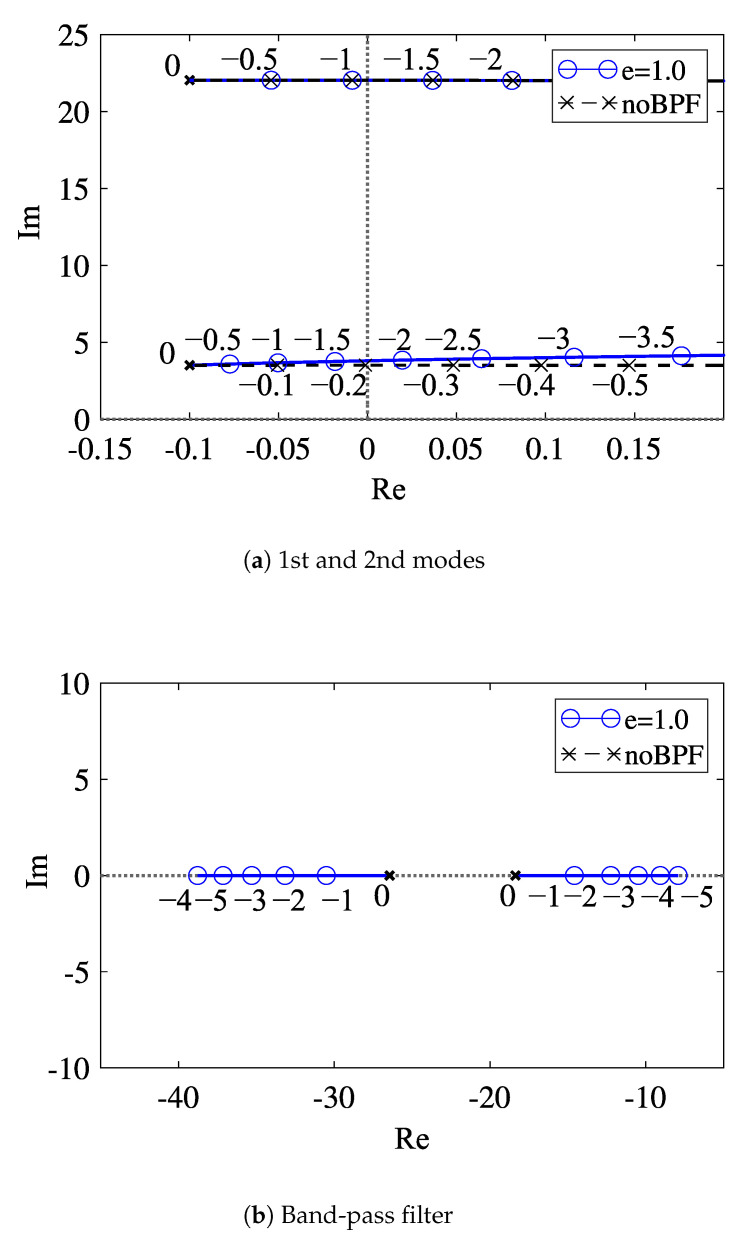
Root loci with respect to the feedback gain $\alpha^{*}$ in the case that the center frequency of the band-pass filter is adjusted to the second mode: (**a**) eigenvalues of the first and second modes; (**b**) eigenvalues of the band-pass filter.

We show the root loci in the case that the center frequency of the band-pass filter is adjusted to the second-mode frequency (e=1) in Figure 3a,b, which describe the shifts of the eigenvalues of the first and second modes and the band-pass filter, respectively.

The solid lines with the round marker in Figure 3 represent the root locus in the case that the band-pass filter is applied to the feedback signal. As seen from Figure 3a, in the case of the band-pass filter whose center frequency is adjusted to the second mode frequency (*e* = 1), the root locus overlaps that in the case without the band-pass filter. The critical feedback gains $\alpha_{2cr}^{*}$ for the second mode are also the same in both cases: $\alpha_{2cr}^{*}=-1.095$. In the case of *e* = 1, the critical feedback gain for the first mode is $\alpha_{1cr}^{*}=-1.752$ as shown in Figure 3a. Because $|\alpha_{1cr}^{*}|$ is larger than $|\alpha_{2cr}^{*}|$, the second mode is self-excited, while the first mode is not destabilized. Furthermore, at the critical feedback gain, the excited frequency is equal to the second natural frequency without a band-pass filter as shown in Figure 3a. Therefore, even in the application of the band-pass filter, we can produce the self-excited oscillation with the natural frequency of the second mode, which is in the case without feedback control. This enables the accurate detection of the frequency shift of the resonator in the sensing.

**Figure 4:**
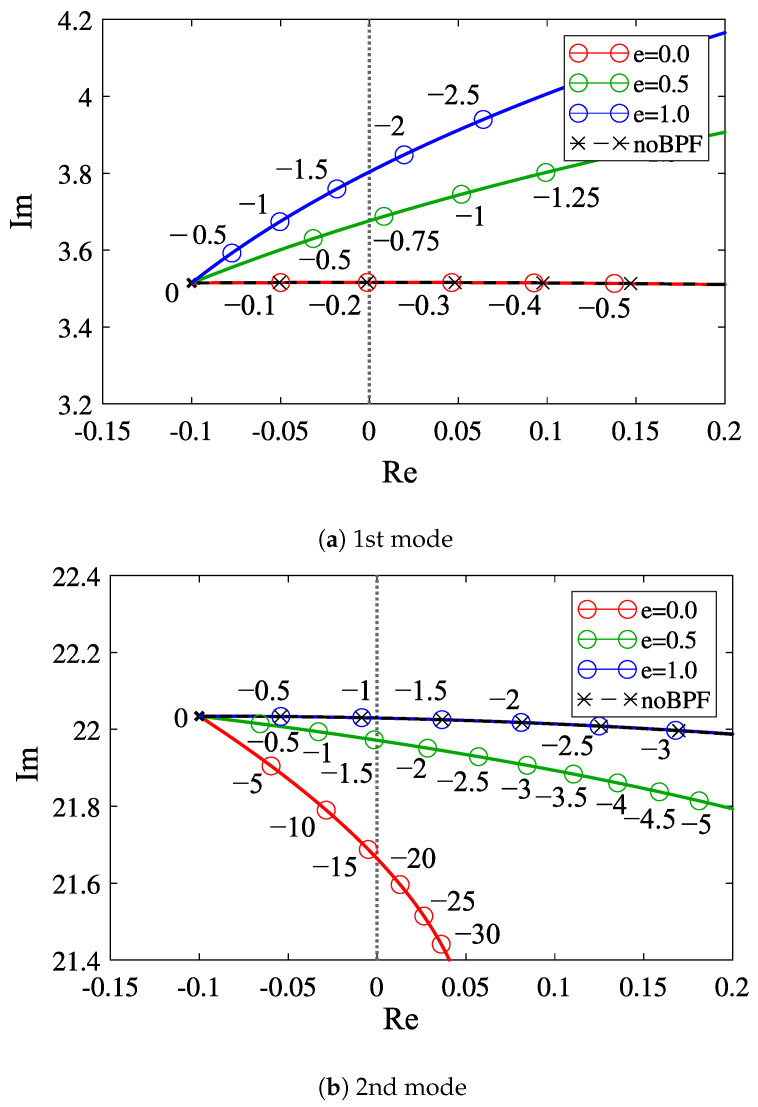
Dependency of the root locus with respect to the feedback gain $\alpha^{*}$ on the center frequency of the band-pass filter; the number denotes the value of the feedback gain: (**a**) first mode; (**b**) second mode.

Figure 4a,b show the variation of the eigenvalues for the first mode and second modes depending on *e*, respectively. The red, green, and blue lines denote those in the cases when the center frequency of the band-pass filter is adjusted to the first-mode frequency (*e* = 0), the middle in the first- and second-mode frequency (*e* = 0.5), and the second-mode frequency (*e* = 1.0), respectively. Figure 4a,b suggest that when the center frequency of the band-pass filter is adjusted to the *i*th mode, the root locus corresponding to the *i*th mode overlaps the root locus in the case without the band-pass filter, and as the center frequency of the band-pass filter is varied from the ith mode frequency, the absolute value of the critical feedback gain becomes larger.

**Table 1:** Parameter values used in the root loci of Figure 4.

Parameter	Value
$\beta^{*}$	1.017
$x_{m}^{*}$	0.73
$\mu^{*}$	0.1

## 3. Experiments for Self-Excited Oscillation in Second Mode Using Microcantilever

### 3.1. Experiment Set-Up

**Figure 5:**
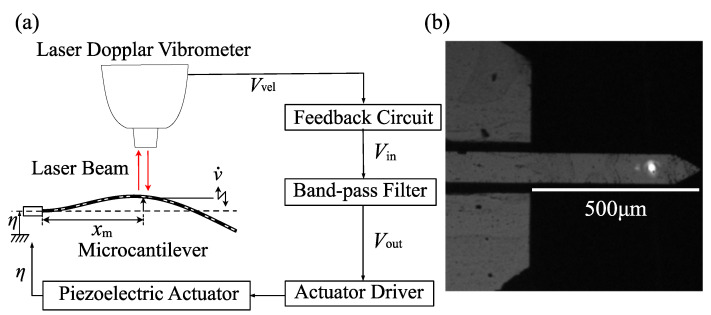
Experimental set-up: (**a**) signal flow in the experiment; (**b**) appearance of the microcantilever.

We performed experiments using a microcantilever to verify the proposed method. Figure 5a,b show, respectively, the schematic diagram of the experimental equipment and the appearance of the silicon microcantilever (ARROW-TL 1 Au-50, Nano World), whose dimensions are 500 μm × 100 μm × 1 μm. According to the frequency response curve of the microcantilever under a frequency sweep, the natural frequencies of the first and second modes are 5.678 kHz and 35.50 kHz, respectively. The velocity of the cantilever measured by the laser Doppler vibrometer (LV-1800, Ono-Sokki; resolution: 0.01 mm/s) is input into the feedback circuit, and the feedback circuit generates the feedback signal expressed as

(20) $$V_{in}=\int \left\{g_{1}\left(\int V_{vel}dt\right)+g_{3}{\left(\int V_{vel}dt\right)}^{3}\right\}dt.$$

**Figure 6:**
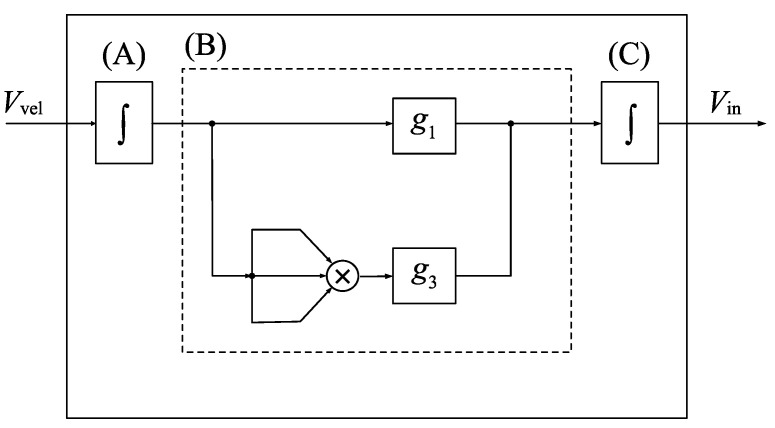
Signal flow in the feedback circuit. The region surrounded by the solid line corresponds to the feedback circuit in Figure 5. (**A**,**C**) denotes an analog integration circuit. The dashed line block (**B**) represents the field-programmable gate array (FPGA) board.

The first term is the linear feedback component to compensate for viscosity and to generate self-excited oscillation. The second term is a nonlinear feedback component to realize the self-excited oscillation with a nontrivial steady-state amplitude by using the dynamics of a van der Pol-type oscillator [20]. The nonlinear effect is theoretically described in Appendix B. The constant coefficients, $g_{1}$ and $g_{3}$, are linear and nonlinear feedback gains, respectively. Figure 6 shows the signal flow in the feedback circuit. The measured velocity signal $V_{vel}$ obtained from the laser Doppler vibrometer is integrated by an analog integral circuit (A) and is input into the field-programmable gate array (FPGA) board (USB-7856R, National Instruments) to calculate the linear and cubic nonlinear feedback components multiplied by the feedback gains $g_{1}$ and $g_{3}$, respectively. The analog integral circuit (C) integrates the output of the FPGA board then the feedback signal $V_{in}$ is output. The FPGA makes it possible to set feedback gains precisely and to output the actuation signal for the control in high processing speed; the cycle time of the FPGA is 2 μs and the resolution of the AD/DA conversion is 16 bit.

The feedback signal is passed through the band-pass filter (3624, NF Corp) and amplified by the piezo driver (ENP-152, Echo Electronics Industry). Then, the signal is input into the piezoelectric actuator (Z1T5×5S-SYXN (C-82); piezoelectric constant: 600 pm/V), which excites the microcantilever. When the response amplitude of the resonator is large, the effects of the nonlinear stiffness and the nonlinear inertia of the microcantilever cannot be neglected [21]. Then, the frequency of the self-excited oscillation is deviated from the natural frequency of the microcantilever depending on the magnitude of the steady-state amplitude [22]. Therefore, in the use of self-excited oscillation, a reduction in the response amplitude is necessary by a special strategy (for example, the application of nonlinear feedback control) [23]. The undesirable shift of the natural frequency due to the temperature variation of the microcantilever does not occur except for the special case using the resonator coated with film [24,25]. However, the temperature variation within the specified operating range of the piezoelectric actuator used in this study is sufficiently small that it does not affect the natural frequency of the cantilever. Therefore, during the experiment, the frequency of the self-excited oscillation is not affected by the temperature.

Through experiments, the nondimensionalized position of the measurement point $x_{m}^{*}$ is 0.73 and the nondimensionalized bandwidth of the band-pass filter $\beta^{*}$ is adjusted to 1.017.

### 3.2. Experimental Results

**Figure 7:**
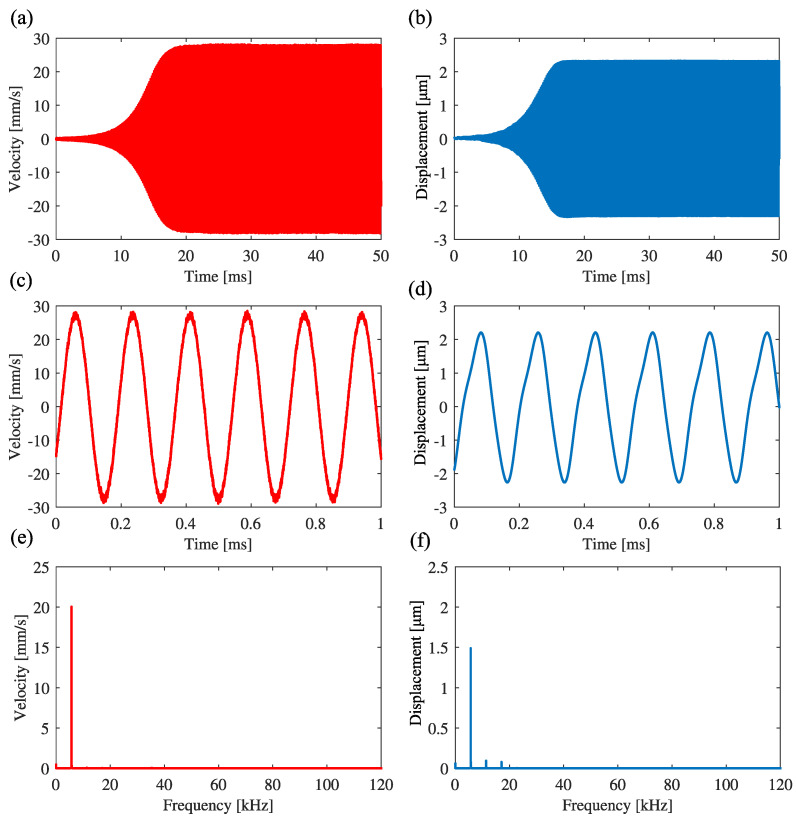
Self-excited oscillation with first mode under feedback control in the case without band-pass filtering: $g_{1}$ = −2.5, $g_{3}$ = 50. (**a**,**b**) are the time histories of the cantilever and the control signal to the piezoelectric actuator. (**c**,**d**) are the expansions of (**a**,**b**) in the steady state, respectively. (**e**,**f**) are the FFT analysis for the oscillation of the beam and the excitation displacement, respectively.

Figure 7 shows the results of the feedback of the signal expressed by Equation (20) to the excitation displacement, where (a) and (b) are the time histories of the velocity signal and the excitation displacement, respectively. Figure 7c,d are the enlarged ones in the steady-state, respectively. Figure 7e,f are the results of FFT analysis of the velocity signal and the excitation displacement, respectively. Because the response frequency is 5.678 kHz in Figure 7e, the system is self-excited with the frequency, which is the natural frequency of the first mode in the case without feedback.

**Figure 8:**
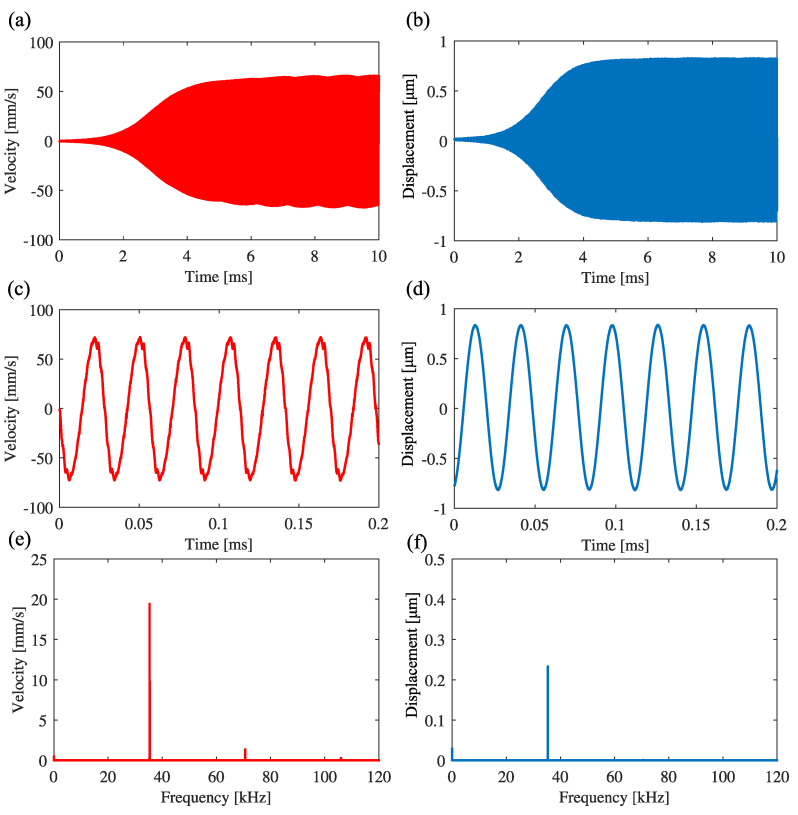
Self-excited oscillation with first mode under feedback control in the case without band-pass filtering: $g_{1}$ = −2, $g_{3}$ = 250. (**a**,**b**) are the time histories of the cantilever and the control signal to the piezoelectric actuator. (**c**,**d**) are the expansions of (**a**,**b**) in the steady state, respectively. (**e**,**f**) are the FFT analysis for the oscillation of the beam and the excitation displacement, respectively.

Figure 8 shows the result of applying the band-pass filter to the signal and feeding back the signal to the excitation displacement, where the measurement point of the velocity signal is the same as that in the experiment in Figure 7. The low-cutoff and high-cutoff frequencies of the band-pass filter are 29.6 kHz and 42.7 kHz, respectively. The center frequency is 35.55 kHz, which is the natural frequency of the second mode. Figure 8a,b are the time histories of the velocity and the excitation displacement, respectively. Figure 8c,d are enlarged ones in the steady state. Figure 8e,f are the results of FFT analysis of the velocity and the excitation displacement, respectively. In Figure 8e, the response frequency of the cantilever is 35.33 kHz, which is near the natural frequency of the second mode. Therefore, it is experimentally confirmed that the utilization of a band-pass filter can produce self-excited oscillation with the second mode.

## 4. Conclusions

Toward a higher-frequency response and more accurate frequency detection in resonators utilized in many microsensors, the utilization of the natural frequency of a higher mode has been focused on. This study proposes a method to produce the self-excited oscillation of a cantilever-type resonator in the second mode by using a band-pass filter. By using the band-pass filter, the feedback signal includes only the natural frequency of the targeted second mode but not that of the first mode. Because the band-pass filter does not generate the phase shift between its input and output signals by suitably adjusting the center frequency, the feedback to compensate for the viscous damping does not affect the response frequency. Therefore, it is possible from the response frequency in the self-excited oscillation to detect the original second natural frequency of the cantilever in the case without feedback. The validity of the proposed method is experimentally confirmed from the self-excited oscillations of a microcantilever. 

## Figures and Tables

**Figure A1:**
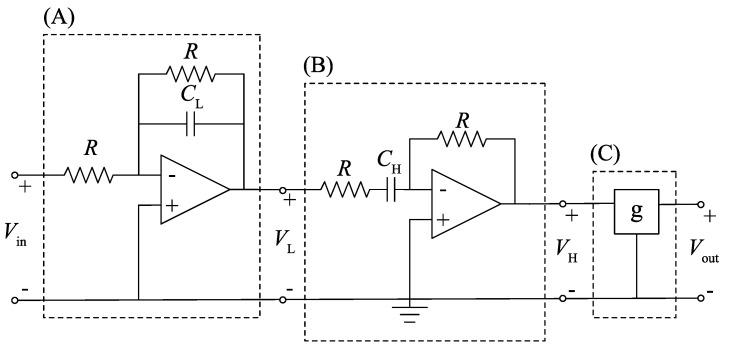
Schematic diagram of an active band-pass filter: (**A**) low-pass filter stage; (**B**) high-pass filter stage; (**C**) noninverting amplifier.

## Data Availability

The data that support the findings of this study are openly available in figshare at https://doi.org/10.6084/m9.figshare.21960023, (access on 26 January 2023).

## Abbreviations

The following abbreviations are used in this manuscript:

FPGA	Field-Programmable Gate Array
FFT	Fast Fourier Translation

## Appendix A

In this appendix, the circuit equation and the transfer function of a band-pass filter are derived. We consider a band-pass filter consisting of a first-order low-pass filter and a first-order high-pass filter connected in the cascade [26] as shown in Figure A1, where the regions surrounded by dashed lines (A), (B), and (C) denote the first-order low-pass filter, the first-order high-pass filter, and the noninverting amplifier, which provides the output gain, respectively.

The input voltage to the band-pass filter is input into the first-order low-pass filter stage (A). According to Kirchhoff’s current law, the relationship between the input voltage $V_{in}$ and the output voltage of the low-pass filter stage $V_{L}$ is expressed as

(A1) $$\frac{1}{R}V_{in}=-\left(\frac{1}{R}V_{L}+C_{L}\frac{dV_{L}}{dt}\right),$$

and the circuit equation for the low-pass filter stage is derived as

(A2) $$\frac{dV_{L}}{dt}+\omega_{L}V_{L}=-\omega_{L}V_{in},$$

where $\omega_{L}=\frac{1}{C_{L}R}$ is the cutoff frequency of the low-pass characteristic of the band-pass filter. Then, the output voltage of the first stage $V_{L}$ is input to the high-pass filter stage (B) and, according to Kirchhoff’s voltage law, the relationship between $V_{L}$ and the output voltage of the high-pass filter stage $V_{H}$ is

(A3) $$\begin{array}{c} \left\{\begin{array}{c} V_{L}-RI-\frac{1}{C_{H}}\int Idt=0\\ -RI-V_{H}=0, \end{array}\right. \end{array}$$

where *I* represents the current through resisters *R* and the capacitor $C_{H}$. The circuit equation of the high-pass filter stage is expressed as

(A4) $$\frac{dV_{H}}{dt}+\omega_{H}V_{H}=-\frac{dV_{L}}{dt},$$

where $\omega_{H}=\frac{1}{C_{H}R}$ is the cutoff frequency of the high-pass characteristic of the band-pass filter. The output voltage of the second stage $V_{H}$ is amplified through the noninverting amplifier (C) and the output voltage of the band-pass filter $V_{out}$ is expressed as

(A5) $$V_{out}=gV_{H},$$

where *g* is the gain of the noninverting amplifier. To construct the band-pass filter as a unity-gain band-pass filter, we set the amplifier gain *g* as follows:

(A6) $$g=\frac{\omega_{L}+\omega_{H}}{\omega_{L}}.$$

Substituting Equations (A2), (A5), and (A6) into (A4) yields the circuit equation of the band-pass filter as

(A7) $$\frac{d^{2}}{dt^{2}}V_{out}+\left(\omega_{L}+\omega_{H}\right)\frac{d}{dt}V_{out}+\omega_{L}\omega_{H}V_{out}=\left(\omega_{L}+\omega_{H}\right)\frac{d}{dt}V_{in}.$$

Considering a phasor input voltage $V_{in}=e^{i\omega t}$, we express the transfer function of the band-pass filter H(ω) as

(A8) $$H\left(\omega \right)=\frac{(\omega_{L}+\omega_{H})i\omega }{\left(\omega_{L}+i\omega \right)\left(\omega_{H}+i\omega \right)}.$$

The magnitude and the phase of the transfer function are expressed as

(A9) $$\left|H\left(\omega \right)\right|=\frac{(\omega_{L}+\omega_{H})\omega }{\sqrt{{\left(\omega_{L}\omega_{H}-\omega^{2}\right)}^{2}+{\left(\omega_{L}+\omega_{H}\right)}^{2}\omega^{2}}},$$

(A10) $$\arg H(\omega )=\arctan\left(\frac{\omega_{L}\omega_{H}-\omega^{2}}{(\omega_{L}+\omega_{H})\omega }\right),$$

respectively [26]. According to Equations (A9) and (A10), when the input frequency ω equals to the center frequency $\omega_{c}$ as

(A11) $$\omega_{c}=\sqrt{\omega_{L}\omega_{H}},$$

the amplitude gain and the phase difference between the input and output voltages are 1 and 0, respectively. Considering the difference between half-power frequencies, the bandwidth of the band-pass filter BW is expressed as

(A12) $$BW=\omega_{L}+\omega_{H}.$$

## Appendix B

We consider the role of the nonlinear feedback component in the experiment, i.e., the second term in Equation (20). The nonlinear feedback is added to Equation (14) as the second term in the following equation:

(A13) $$V_{in}=g_{in}\int {v|}_{x=x_{m}}dt+g_{in3}\int {\left(v{|}_{x=x_{m}}\right)}^{3}dt,$$

where the first term is the linear feedback corresponding to the left-hand side in Equation (14) and $g_{in3}$ is the nonlinear feedback gain. Substituting Equation (A13) into Equations (1), (15), and (16) and nondimensionalizing the result as in Section 2 yields the nondimensionalized equation of motion of the cantilever under the linear and nonlinear feedback as

(A14) $$\begin{array}{c} \left\{\begin{array}{c} {\ddot{v}}^{*}+2\mu^{*}{\dot{v}}^{*}+v^{*\prime \prime \prime \prime }=2\beta^{*}\omega_{c}^{*}{\dot{V}}_{out}^{*}+\omega_{c}^{*2}V_{out}^{*}-2\beta^{*}\omega_{c}^{*}\left\{\alpha^{*}v^{*}{|}_{x^{*}=x_{m}^{*}}+\alpha_{3}^{*}{\left(v^{*}{|}_{x^{*}=x_{m}^{*}}\right)}^{3}\right\}\\ {\ddot{V}}_{out}^{*}+2\beta^{*}\omega_{c}^{*}{\dot{V}}_{out}^{*}+\omega_{c}^{*2}V_{out}^{*}=2\beta^{*}\omega_{c}^{*}\left\{\alpha^{*}v^{*}{|}_{x^{*}=x_{m}^{*}}+\alpha_{3}^{*}{\left(v^{*}{|}_{x^{*}=x_{m}^{*}}\right)}^{3}\right\}, \end{array}\right. \end{array}$$

where $\alpha_{3}^{*}=g_{actuator}g_{in3}L^{2}T$ is the nondimensionalized nonlinear feedback gain. Applying mode decomposition to Equation (A14) using Equations (5)–(7) yields the equation of state as

(A15) $$\begin{array}{cc} \frac{d}{dt^{*}}\left[\begin{array}{c} v_{1}^{*}\\ {\dot{v}}_{1}^{*}\\ v_{2}^{*}\\ {\dot{v}}_{2}^{*}\\ V_{out}^{*}\\ {\dot{V}}_{out}^{*} \end{array}\right]= & \left[\begin{array}{cccc} 0 & 1 & 0 & \\ -\omega_{1}^{*}-2\beta^{*}\omega_{c}^{*}\alpha^{*}a_{1}^{*} & -2\mu^{*} & -2\beta^{*}\omega_{c}^{*}\alpha^{*}a_{2}^{*} & \\ 0 & 0 & 0 & \\ -2\beta^{*}\omega_{c}^{*}\alpha^{*}a_{1}^{*} & 0 & -\omega_{2}^{*}-2\beta^{*}\omega_{c}^{*}\alpha^{*}a_{2}^{*} & *\\ 0 & 0 & 0 & \\ 2\beta^{*}\omega_{c}^{*}\alpha^{*}a_{1}^{*} & 0 & 2\beta^{*}\omega_{c}^{*}\alpha^{*}a_{2}^{*} & \end{array}\right.\\ \; & \left.\begin{array}{cccc} \; & 0 & 0 & 0\\ \; & 0 & {\omega_{c}^{*}}^{2} & 2\beta^{*}\omega_{c}^{*}\\ \; & 1 & 0 & 0\\ {}* & -2\mu^{*} & {\omega_{c}^{*}}^{2} & 2\beta^{*}\omega_{c}^{*}\\ \; & 0 & 0 & 1\\ \; & 0 & -{\omega_{c}^{*}}^{2} & -2\beta^{*}\omega_{c}^{*} \end{array}\right]\left[\begin{array}{c} v_{1}^{*}\\ {\dot{v}}_{1}^{*}\\ v_{2}^{*}\\ {\dot{v}}_{2}^{*}\\ V_{out}^{*}\\ {\dot{V}}_{out}^{*} \end{array}\right]+2\beta^{*}\omega_{c}^{*}\alpha_{3}^{*}{\left(a_{1}^{*}v_{1}^{*}+a_{2}^{*}v_{2}^{*}\right)}^{3}\left[\begin{array}{c} 0\\ 1\\ 0\\ 1\\ 0\\ 1 \end{array}\right]. \end{array}$$

As seen from the root locus in Figure 3a,b, when linear feedback gain $\alpha^{*}$ equals the critical feedback gain of the second mode $\alpha_{2cr}^{*}$, the eigenvalues of the coefficient matrix of the linear part can be expressed as

(A16) $$\lambda =-\gamma_{1}\pm i{\omega_{1}^{*}}^{\prime },\pm i\omega_{2}^{*},-\gamma_{31},-\gamma_{32},$$

where $-\gamma_{1}\pm i{\omega_{1}^{*}}^{\prime },\pm i\omega_{2}^{*}$ are the eigenvalues shifted from those of the first and second modes of the cantilever, respectively, and $-\gamma_{31}$ and $-\gamma_{32}$ are the eigenvalues corresponding to the poles of the band-pass filter, respectively. This matrix can be transformed into a block diagonal form by a transformation matrix *P* as [27]

(A17) $$\frac{d}{dt^{*}}\left[\begin{array}{c} y_{1}^{*}\\ y_{2}^{*}\\ y_{3}^{*}\\ y_{4}^{*}\\ y_{5}^{*}\\ y_{6}^{*} \end{array}\right]=\left[\begin{array}{cccccc} -\gamma_{1}^{*} & {\omega_{1}^{*}}^{\prime } & 0 & 0 & 0 & 0\\ -{\omega_{1}^{*}}^{\prime } & -\gamma_{1}^{*} & 0 & 0 & 0 & 0\\ 0 & 0 & 0 & \omega_{2}^{*} & 0 & 0\\ 0 & 0 & -\omega_{2}^{*} & 0 & 0 & 0\\ 0 & 0 & 0 & 0 & -\gamma_{31}^{*} & 0\\ 0 & 0 & 0 & 0 & 0 & -\gamma_{32}^{*} \end{array}\right]\left[\begin{array}{c} y_{1}^{*}\\ y_{2}^{*}\\ y_{3}^{*}\\ y_{4}^{*}\\ y_{5}^{*}\\ y_{6}^{*} \end{array}\right]+\alpha_{3}^{*}F,$$

where the transformed state vector ${\left[\begin{array}{cccccc} y_{1}^{*} & y_{2}^{*} & y_{3}^{*} & y_{4}^{*} & y_{5}^{*} & y_{6}^{*} \end{array}\right]}^{t}$ is defined as

(A18) $$\left[\begin{array}{c} y_{1}^{*}\\ y_{2}^{*}\\ y_{3}^{*}\\ y_{4}^{*}\\ y_{5}^{*}\\ y_{6}^{*} \end{array}\right]=P^{-1}\left[\begin{array}{c} v_{1}^{*}\\ {\dot{v}}_{1}^{*}\\ v_{2}^{*}\\ {\dot{v}}_{2}^{*}\\ V_{out}^{*}\\ {\dot{V}}_{out}^{*} \end{array}\right],$$

and F is the transformed nonlinear feedback component as

(A19) $$F=\left[\begin{array}{c} {\left(f_{11}y_{1}^{*}+f_{12}y_{2}^{*}+f_{13}y_{3}^{*}+f_{14}y_{4}^{*}+f_{15}y_{5}^{*}+f_{16}y_{6}^{*}\right)}^{3}\\ \vdots \\ {\left(f_{61}y_{1}^{*}+f_{62}y_{2}^{*}+f_{63}y_{3}^{*}+f_{64}y_{4}^{*}+f_{65}y_{5}^{*}+f_{66}y_{6}^{*}\right)}^{3} \end{array}\right],$$

where the coefficients $f_{ij}$ depend on the nondimensionalized parameters β and μ. Considering that the eigenvalues corresponding to the first mode of the cantilever and to the poles of the band-pass filter are located in the left-half plane of the complex plane, the dynamics of $y_{1}^{*}$, $y_{3}^{*}$, $y_{5}^{*}$, and $y_{6}^{*}$ decay rapidly. Therefore, the dynamics of the system including the band-pass filter are governed by only $y_{3}^{*}$ and $y_{4}^{*}$. To investigate the situation when the linear feedback gain is in the neighborhood of the critical one of the second mode, we introduce the small parameter ε and express $\alpha^{*}$ as $\alpha^{*}=\alpha_{2cr}^{*}-\alpha \alpha^{*}\;\;\left(\alpha \alpha^{*}=O\left(\varepsilon \right),0<\varepsilon <<1\right)$. Then, the dynamics of $y_{3}^{*}$ and $y_{4}^{*}$ are governed by

(A20) $$\frac{d}{dt^{*}}\left[\begin{array}{c} y_{3}^{*}\\ y_{4}^{*} \end{array}\right]=\left[\begin{array}{cc} \gamma_{2}^{*} & \omega_{2}^{*}\\ -\omega_{2}^{*} & \gamma_{2}^{*} \end{array}\right]+\alpha_{3}^{*}\left[\begin{array}{c} g_{30}{y_{3}^{*}}^{3}+g_{21}{y_{3}^{*}}^{2}y_{4}^{*}+g_{12}y_{3}^{*}{y_{4}^{*}}^{2}+g_{03}{y_{4}^{*}}^{3}+\cdots \\ h_{30}{y_{3}^{*}}^{3}+h_{21}{y_{3}^{*}}^{2}y_{4}^{*}+h_{12}y_{3}^{*}{y_{4}^{*}}^{2}+h_{03}{y_{4}^{*}}^{3}+\cdots \end{array}\right],$$

where $\gamma_{2}^{*}$ related to $\alpha \alpha^{*}$ is a small parameter and can be set $\gamma_{2}^{*}=\varepsilon \hat{\gamma_{2}^{*}}\;\;\left(\hat{\gamma_{2}^{*}}=O\left(1\right)\right)$, and the coefficients $g_{ij}$ and $h_{ij}$ are calculated from Equation (A19). By introducing *z* as

(A21) $$z=iy_{3}^{*}+y_{4}^{*},$$

Equation (A20) is written as

(A22) $$\frac{d}{dt^{*}}z=\left(\varepsilon \hat{\gamma_{2}^{*}}+i\omega_{2}^{*}\right)z+\alpha_{3}^{*}F{\left|z\right|}^{2}z+\mathrm{NST},$$

where NST denotes the nonsecular term and *F* is the complex coefficient of the cubic nonlinear term. Equation (A22) is rewritten as

(A23) $$\frac{d}{dt^{*}}a=2\varepsilon \hat{\gamma_{2}^{*}}a+2\alpha_{3}^{*}F_{r}a^{2},$$

where $a={\left|z\right|}^{2}$ corresponds to the square of the oscillation amplitude and $F_{r}$ is the real part of *F*. Then, the conditions that a stable nontrivial steady-state amplitude exists are as follows:

(A24) $$\left\{\begin{array}{c} \varepsilon \hat{\gamma_{2}^{*}}>0\\ \alpha_{3}^{*}F_{r}<0. \end{array}\right.$$

Under this condition, the self-excited oscillation has the steady-state amplitude $\sqrt{-\frac{\varepsilon \hat{\gamma_{2}^{*}}}{\alpha_{3}^{*}F_{r}}}$. Therefore, we can freely set the magnitude by changing the nonlinear feedback gain $\alpha_{3}^{*}$.
